# Neurological manifestations in speech after snake bite: a rare case

**DOI:** 10.4314/pamj.v4i1.53597

**Published:** 2010-03-24

**Authors:** Dharam Vir, Sachin, Dipti Gupta, Munish Modi, Naresh Panda

**Affiliations:** 1 Department of Otolaryngology and Head and Neck Surgery, Post Graduate Institute of Medical Education and Research, Chandigarh, India;; 2 Department of Neurology, Post Graduate Institute of Medical Education and Research, Chandigarh, India

**Keywords:** Snake bite, speech therapy, flaccid dysarthria, dysarthria

## Abstract

The patient was admitted after reporting a snake bite from which he later developed neurological signs and symptoms among which a flaccid dysarthria. The patient underwent speech therapy and showed significant improvement over a short period of time. The favorable outcome of the present study highlights the role of speech therapy in such a case, where it often remains un-emphasized.

## Patient and case report

A 45-year old male was bitten by a snake on the left finger while sleeping. Immediately after snake bite patient reported difficulty in breathing. He was taken to a nearby local civil hospital where antivenom management for snake bite was provided.

After three hours of clinical observation, the breathing condition of the patient did not improve. He was transferred to the Emergency Department of the Post Graduate Institute of Medical Education and Research (PGIMER) where he arrived approximately 5 hours after the snake bite. On arrival, the patient was conscious and responded to verbal commands; pupils were dilated, but reactive to light; blood pressure was 180/100 mmHg; pulse rate was 100/min; ptosis was present in both the eyes accompanied by weakness in eye muscles. The patient was kept on mechanical ventilator for next 3 days.

Two days after weaning of ventilator support, he developed numbness in all the extremities. Neurological examination revealed hypotonia, weakness in the neck, jaw and respiratory muscles. Motor power was grade 1.

18 hours after snakebite the patient was stuporous. 20 hours after the snakebite he went into a state of coma. He had no response to stimuli, fixed dilated pupil at 5 mm and full ptosis. The patient received 3 vials of specific antivenom for Bungarus candidus because nocturnal bites causing neurotoxic signs and symptoms without local necrosis are typical of envenoming bites by kraits and B. candidus; snakes which are common in the area in which the incident happened. Thirty hours after snakebite, the patient responded to verbal commands, while his motor power improved to grade 1. Forty hours after the snakebite and 32 vials of specific antivenom, motor power had improved to grade 4. He could open his eyelids easily but slurring in speech was present. The patient was finally he was discharged from the hospital ten days after the snake bite. The patient was referred to Speech and Hearing Unit, attached to the Department of Otolaryngology, PGIMER, Chandigarh immediately after being discharged from the medical OPD, with a chief complaint of slurring of speech, resonance problem, reduced loudness and misarticulations. These complaints were documented by the patient immediately when his consciousness had improved to verbal output.

## Speech assessment

Franchay’s Dysarthria Assessment tool [1] was administered on the patient. Detailed assessment revealed that patient had flaccid dysarthria. The general characteristics found included slurring of speech, moderate hyper nasality, hypotonia and lowered didochokinetic rate. There was significant nasal air emission. His breathing pattern was thoracic-clavicular. Tongue movements and mouth opening were restricted. Struggle behavior was evident in connected speech. His loudness was reduced. Dynamic range of pitch and intensity of voice was also found to be reduced. Frequent gaps of involuntary silence were observed while conversing with the patient. Paresis and atrophy of musculature, fibrillations and fasciculations were also evident during the oro-musculature examination. EMG studies were conducted and 4 paired muscles were recorded because of their antagonistic functions: 1) Orbicularis oris inferioris and depressor labii inferioris, 2) mentalis and buccinators. The findings revealed disturbed firing patterns and well defined abnormalities. These speech symptoms could be attributed to neurological insult by toxic snake bite.

After reviewing clinical, neurological and speech findings, a diagnosis of Flaccid Dysarthria was made which is a rare entity on the account of neurotoxic snake bite.

## Management

The patient was counseled regarding the nature of problem and prognosis. He was scheduled for daily speech therapy of 1 hour duration for 3 months. Open mouth approach was advised to improve his loudness. His speech was worked upon vigorously to improve his intelligibility. Specific concerns included breathing pattern changes, limited speech intelligibility due to slurring of speech, attempts to improve the didochokinetic rate and intensity. There was a considerable improvement of the patient’s speech and the didochokinetic rate of the patient improved to 1.5–2.0 per second, which is grossly normal. The favorable outcome of the present study provides an important example of the beneficial effects of therapy even for clients with anticipated fair prognosis. Specific antivenom for B. Candidus, antibiotic drugs, antihypertensive drugs and tetanus toxoid were given to the patient as autonomic functions such as tachycardia, and hypertension were observed. The patient received specific antivenom for Bungarus Candidus after being bitten at 10 hours respectively. The first clinical response in this case was observed 20 hours after receiving 16 vials; this was in the form of slight movement of fleet phalanxes. At 40 hours after receiving 32 vials of specific antivenom he was able to respond to verbal commands, motor power changed from grade1 to grade 4 with 100% elevation of eyebrows from full ptosis. The patient had spontaneous opening of eyelids at 90 hours after receiving specific antivenom therapy. The patient was discharged later on day ten.

## Discussion

This case was found worth reporting as it was a very rare occurrence. Similar cases probably go unnoticed as the primary concern is basic physical recovery, speech is a later concern. Although many studies were found in European and American countries [2] but very few studies are presented in Indian literature, that too from speech perspective [3]. Although the recovery rate is high in those patients who received specific antivenom therapy, the speech symptoms often persist. Speech problems have been considered at secondary level and not given equal importance. The patient has been coming for regular speech therapy for the last three months and there has been a marked improvement in his articulation and loudness. Moreover, the overall speech intelligibility of the patient has improved to a great extent. Even though the paresis of lower motor neurons, motor and respiratory muscles, neurotoxicity of the venom include the autonomic dysfunctions, the patient showed tremendous improvement in his speech and articulatory proficiency.

## Conclusion

Returning of near normal speech after receiving speech therapy showed the importance of timely speech therapy intervention. Clinical response includes returning of good speech with improved loudness and intelligibility.

## Conflict of interest

The authors declared they have no competing interests.

## Authors’ contribution

**DV** helped in diagnosing and managing the speech problems of the patient. He contributed to the write up of the manuscript. **S** worked on the case and managed it with his full expertise. Collaborated with the neurology department and collected all information necessary for preparing the manuscript. **DG** gave suggestions regarding the various neurological aspects of speech and helped in writing the manuscript. **MM** provided all the necessary information as far as neurological aspects were concerned in this case. Moreover, he reviewed the final manuscript and gave his expert comments. **NP** provided all the necessary information as far as laryngologic aspects were concerned in this case. Moreover, he reviewed the final manuscript and gave his expert suggestions.

## Acknowledgement

Permission was taken from the patient for writing this case. We highly acknowledge his gesture.
